# Influence of the Position and Composition of Radiometals and Radioiodine Labels on Imaging of Epcam Expression in Prostate Cancer Model Using the DARPin Ec1

**DOI:** 10.3390/cancers13143589

**Published:** 2021-07-17

**Authors:** Sergey M. Deyev, Tianqi Xu, Yongsheng Liu, Alexey Schulga, Elena Konovalova, Javad Garousi, Sara S. Rinne, Maria Larkina, Haozhong Ding, Torbjörn Gräslund, Anna Orlova, Vladimir Tolmachev, Anzhelika Vorobyeva

**Affiliations:** 1 Research Centrum for Oncotheranostics, Research School of Chemistry and Applied Biomedical Sciences, Tomsk Polytechnic University, 634050 Tomsk, Russia; biomem@mail.ru (S.M.D.); schulga@gmail.com (A.S.); marialarkina@mail.ru (M.L.); anna.orlova@ilk.uu.se (A.O.); anzhelika.vorobyeva@igp.uu.se (A.V.); 2 Molecular Immunology Laboratory, Shemyakin & Ovchinnikov Institute of Bioorganic Chemistry, Russian Academy of Sciences, 117997 Moscow, Russia; Elena.ko.mail@gmail.com; 3 Bio-Nanophotonic Lab., Institute of Engineering Physics for Biomedicine (PhysBio), National Research Nuclear University “MEPhI”, 115409 Moscow, Russia; 4 Department of Immunology, Genetics and Pathology, Uppsala University, 751 85 Uppsala, Sweden; tianqi.xu@igp.uu.se (T.X.); yongsheng.liu@igp.uu.se (Y.L.); garousi@kth.se (J.G.); 5 Department of Protein Science, School of Engineering Sciences in Chemistry, Biotechnology and Health, KTH Royal Institute of Technology, 114 17 Stockholm, Sweden; haozhong@kth.se (H.D.); torbjorn@kth.se (T.G.); 6 Department of Medicinal Chemistry, Uppsala University, 751 23 Uppsala, Sweden; sara.rinne@ilk.uu.se; 7 Department of Pharmaceutical Analysis, Siberian State Medical University (SSMU), 2, Moscow Trakt, 634050 Tomsk, Russia; 8 Science for Life Laboratory, Uppsala University, 751 23 Uppsala, Sweden

**Keywords:** radionuclide, EpCAM, DARPin, PET, SPECT, imaging, prostate, cancer

## Abstract

**Simple Summary:**

Metastasis-targeting therapy might improve outcomes in oligometastatic prostate cancer. Epithelial cell adhesion molecule (EpCAM) is overexpressed in 40–60% of prostate cancer cases and might be used as a target for specific delivery of toxins and drugs. Radionuclide molecular imaging could enable non-invasive detection of EpCAM and stratification of patients for targeted therapy. Designed ankyrin repeat proteins (DARPins) are scaffold proteins, which can be selected for specific binding to different targets. The DARPin Ec1 binds strongly to EpCAM. To determine an optimal design of Ec1-based probes, we labeled Ec1 at two different positions with four different nuclides (^68^Ga, ^111^In, ^57^Co and ^125^I) and investigated the impact on Ec1 biodistribution. We found that the *C*-terminus is the best position for labeling and that ^111^In and ^125^I provide the best imaging contrast. This study might be helpful for scientists developing imaging probes based on scaffold proteins.

**Abstract:**

The epithelial cell adhesion molecule (EpCAM) is intensively overexpressed in 40–60% of prostate cancer (PCa) cases and can be used as a target for the delivery of drugs and toxins. The designed ankyrin repeat protein (DARPin) Ec1 has a high affinity to EpCAM (68 pM) and a small size (18 kDa). Radiolabeled Ec1 might be used as a companion diagnostic for the selection of PCa patients for therapy. The study aimed to investigate the influence of radiolabel position (*N*- or *C*-terminal) and composition on the targeting and imaging properties of Ec1. Two variants, having an *N*- or *C*-terminal cysteine, were produced, site-specifically conjugated to a DOTA chelator and labeled with cobalt-57, gallium-68 or indium-111. Site-specific radioiodination was performed using ((4-hydroxyphenyl)-ethyl)maleimide (HPEM). Biodistribution of eight radiolabeled Ec1-probes was measured in nude mice bearing PCa DU145 xenografts. In all cases, positioning of a label at the *C*-terminus provided the best tumor-to-organ ratios. The non-residualizing [^125^I]I-HPEM label provided the highest tumor-to-muscle and tumor-to-bone ratios and is more suitable for EpCAM imaging in early-stage PCa. Among the radiometals, indium-111 provided the highest tumor-to-blood, tumor-to-lung and tumor-to-liver ratios and could be used at late-stage PCa. In conclusion, label position and composition are important for the DARPin Ec1.

## 1. Introduction

Androgen deprivation therapy (in combination with abiraterone or docetaxel) is the most efficient treatment for metastatic prostate cancer. However, metastasis-targeting therapy might improve the outcome for patients with oligometastatic prostate cancer [1,2]. An increasing amount of clinical evidence suggests that radionuclide therapy using prostate-specific membrane antigen (PSMA) ligands could be developed into a powerful tool for the treatment of metastases of prostate cancer [3,4]. Still, up to 24% of patients with metastatic prostate cancer have too-low PSMA expression and are not eligible for PSMA-targeted treatment [5]. Therefore, a complementary targeted therapy is needed for these patients.

A possible therapeutic target for targeted therapy of prostate cancer might be the epithelial cell adhesion molecule (EpCAM). EpCAM is appreciably overexpressed in local and metastatic prostate cancer (PCa) compared to non-cancerous prostate cells and is a prognostic biomarker for a poor patient outcome [6,7]. Induction of EpCAM expression occurs early in prostate carcinogenesis and the expression increases with Gleason score and tumor stage [6]. EpCAM overexpression in PCa is associated with metastasis, resistance to chemo- and radiotherapy [8,9] and an increased risk of cancer recurrence [7,10], which makes it a promising therapeutic target.

Several EpCAM-targeting therapeutics are in pre-clinical and clinical development [11,12,13,14,15,16]. It would be attractive to apply such targeted drugs for metastases-targeted therapy of oligometastatic prostate cancer. However, such therapeutics often contain potent targeting toxins or cytotoxic drugs, which might have severe side effects. Apparently, their application is only justified if a potential benefit outweighs the potential risks. The anti-tumor effect of such therapeutics depends on the target expression level. An intense EpCAM overexpression is observed in 40–60% of collected tumor samples [17], and a reliable stratification method is thus required for a successful clinical application of the targeted therapies. 

Radionuclide molecular imaging of therapeutic targets is considered a powerful tool for the stratification of patients for targeting therapies [18]. This technique has the potential to solve the major inherent drawbacks of biopsy-based stratification methods, invasiveness and target expression heterogeneity, leading to non-representative sampling. An important precondition for the application of molecular imaging for stratification is high sensitivity. This is essential because in oligometastatic disease, small lymph node metastases would be a typical form of the disease.

Experience with different classes of targeting probes for radionuclide imaging suggests that engineered scaffold proteins (ESP) are one of the most attractive formats [19,20]. ESPs are based on non-immunoglobulin scaffolds, which permit substantial reduction in probe size, down to 5–20 kDa. The small size facilitates prompt extravasation and diffusion in the extracellular space of the tumors, enabling quick (within less than 30 min) accumulation in tumors. Furthermore, the non-bound tracers are rapidly excreted via kidneys, which favors a high imaging contrast.

A possible candidate as a targeting probe for EpCAM imaging is the designed ankyrin repeat protein (DARPin) Ec1 [21]. The DARPin scaffold contains four or five repeat modules and has a molecular weight of 14 to 18 kDa [22]. The DARPin scaffold has been found to allow for the generation of binders with a very high affinity to selected molecular targets. The five-repeats DARPin Ec1 (MW 18.6 kDa) binds to EpCAM with an affinity of 60 pM [21]. Radiolabeled Ec1 derivatives have earlier been successfully used for imaging of EpCAM-expressing pancreatic, ovarian and triple-negative breast cancers [23,24,25]. It has to be noted that the requirements for the development of imaging probes for oligometastatic prostate cancer are different from the requirements for probes for imaging of ovarian, pancreatic and breast cancers. Metastases of these cancers are frequently located in the liver, the peritoneal organs and the lungs [26]. Therefore, in these cases, the imaging probes should provide a high contrast with these organs and tissues. Hepatic uptake and hepatobiliary excretion for such tracers are therefore utterly undesirable as they result in high background and low contrast in the organs of interest. 

Conversely, oligometastatic prostate cancer is confined in the pelvis. Therefore, the highest priority is to provide the highest possible contrast to tissues constituting an anatomical context for this cancer, i.e., muscle, bone and blood. Hepatobiliary excretion is not a problem in this case, and it might be even desirable since it minimizes activity in the urinary bladder. 

Unlike antibodies, ESPs are relatively easy to engineer. This enables, for example, the incorporation of single cysteines at desired positions of cysteine-free ESP scaffolds. This creates a unique sulfhydryl group in the whole structure. Chelators or prosthetic groups can be coupled to this group site-specifically using thiol-reactive constituents, e.g., maleimido derivatives. This provides well-defined conjugates with reproducible biologic properties [27]. Previous experience with affibody molecules shows that selection of an optimal labeling strategy, i.e., the combination of a radionuclide, chelator or linker for its attachment and position of a label on the ESP, provides an opportunity to create an imaging agent with desirable properties [20,27]. The general complexity of in vivo interactions of proteins does not allow the prediction of the optimal structure of an imaging probe. This necessitates structure–properties studies in vivo. 

**Figure 1:**
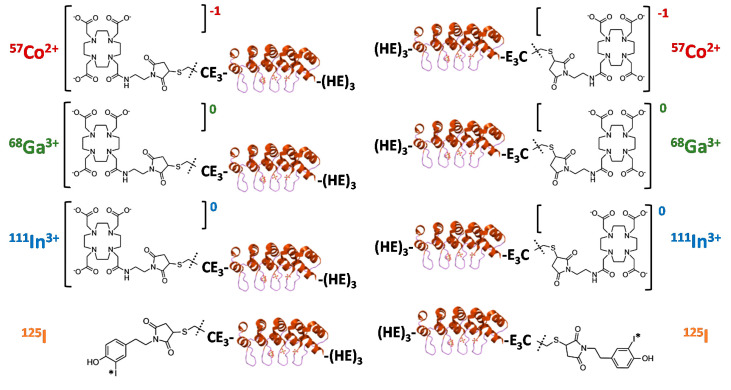
Schematic overview of the structures of Ec1 variants radiolabeled with cobalt-57, gallium-68, indium-111 or iodine-125 at the *N*- or *C*-terminus.

The goal of this study was to evaluate the biodistribution and targeting pattern of a panel of Ec1 DARPins labeled with different nuclides (Figure 1). Different positions of labels (*N*- or *C*-terminus) were included based on experience with the anti-HER2 DARPin G3 labeled using [^99m^Tc]Tc(CO)_3_, which suggested that the label position has a strong effect on biodistribution [28]. ^68^Ga (T _½_ = 67.6 min) was selected as a positron emitter, which might be readily available using a ^68^Ge/^68^Ga generator. ^111^In (T _½_ = 2.8 d) is a commonly used radionuclide for SPECT. ^57^Co (T _½_ = 272 d) was used as a convenient surrogate for the long-lived positron emitter ^55^Co (T _½_ = 17.5 h). The use of ^55^Co might be helpful if delayed imaging (at 24 h after injection) provides better contrast than imaging during the day of injection [29]. The versatile DOTA chelator was selected for labeling since it allows for stable coupling of the selected metals [30]. ^125^I (T _½_ = 59.4 d) was chosen as a convenient surrogate for ^123^I (T _½_ = 13.3 d) and ^124^I (T _½_ = 4.18 d), which are used for imaging using SPECT and PET, respectively. The radioiodination was performed using a HPEM linker because this approach provides site-specific coupling radioiodine to proteins. For the anti-HER2 DARPin G3, such labeling was associated with elevated hepatobiliary excretion [31], which might be an advantage in the case of imaging of oligometastatic prostate cancer.

## 2. Materials and Methods

### 2.1. General Materials and Instruments

The radionuclides Na [^125^I]I and [^57^Co]CoCl_2_ were purchased from Perkin Elmer Sverige AB (Sweden); [^111^In]InCl_3_ was purchased from Curium Pharma (Curium Netherlands B.V., Petten, The Netherlands). Gallium-68 was eluted from a ^68^Ga/^68^Ge generator (Cyclotron Co., Obninsk, Russia) with 0.1 M HCl. The buffers used for labeling were prepared using Milli-Q water and were treated with Chelex 100 resin (Bio-Rad Laboratories, Richmond, VA, USA) to remove metal contamination. Maleimido-mono-amide-DOTA was purchased from Macrocyclics (Plano, TX, USA). Instant thin-layer chromatography (iTLC) analysis was performed using iTLC silica gel strips (Varian, Lake Forest, CA, USA). The activity distribution was measured using a Cyclone storage phosphor system and was analyzed by OptiQuant image analysis software (both from PerkinElmer, Waltham, MA, USA). Purification was performed using NAP-5 size-exclusion columns (GE Healthcare, UK). PC-3, DU145 and Ramos cells were purchased from the American Type Culture Collection (ATCC, Manassas, VA, USA) and were cultured in RPMI medium supplemented with fetal bovine serum (FBS) (20% for PC-3 cells, 10% for DU145 and Ramos cells), 2 mM l-glutamine, 100 IU/mL penicillin and 100 µg/mL streptomycin in a humidified incubator with 5% CO_2_ at 37 °C, unless indicated otherwise. The activity was measured by an automated gamma-spectrometer with NaI(Tl) detector (2480 Wizard, Wallac, Turku, Finland).

### 2.2. Protein Production and Conjugation to DOTA

Two Ec1 variants were produced based on the published sequence [21] using methodology described earlier [32]. Purification was performed using a combination of immobilized metal ion chromatography (IMAC) and ion-exchange chromatography. One Ec1 variant had a cysteine followed by three glutamates at the N-terminus and a histidine–glutamate (HE)_3_ tag at *C*-terminus (CE_3_-Ec1-(HE)_3_). The other Ec1 variant had a histidine–glutamate (HE)_3_ tag at N-terminus and three glutamates followed by a cysteine at the *C*-terminus ((HE)_3_-Ec1-E_3_C). The molecular weight of the DARPins was measured by liquid chromatography–electrospray ionization–mass spectrometry (LC–ESI–MS) on a 6520 Accurate Q-TOF LC/MS (Agilent, Santa Clara, CA, USA).

For conjugation of Ec1 variants to maleimide-DOTA, CE_3_-Ec1-(HE)_3_ or (HE)_3_-Ec1-E_3_C (0.65 mg, 35 nmoles, 464 µL in PBS, pH 7.4) was first incubated with a 280-fold molar excess of dithiothreitol (DTT) (1.5 mg, 9.7 µmoles, 30 µL in PBS, pH 8.0), final DTT concentration 20 mM, for 30 min at 40 °C. To remove DTT, the reaction mixture was purified using a NAP-5 column, pre-equilibrated with degassed 0.2 M ammonium acetate (NH_4_OAc), pH 6.5. Next, the fractions containing the protein (0.6 mg, 32 nmoles, 470 µL) were incubated with an 11-fold molar excess of maleimide-DOTA (275 µg, 350 nmoles, 28 µL of 10 mg/mL in 0.2 M NH_4_OAc, pH 6.5) at 40 °C for 1 h. To remove the unconjugated chelator, the reaction mixture was purified using a NAP-5 column with 0.2 M NH_4_OAc, pH 6.5. The protein concentration was measured using a DS-11 spectrophotometer (DeNovix, Wilmington, DE, USA). The CE_3_-Ec1-(HE)_3_ and (HE)_3_-Ec1-E_3_C variants conjugated to DOTA (termed DOTA–Ec1 and Ec1–DOTA, respectively) were stored in 0.2 M NH_4_OAc (pH 6.5) at −20 °C before labeling with radiometals.

### 2.3. Radiolabeling and Stability

Site-specific labeling of Ec1 variants with iodine-125 was performed in three steps using a maleimide–cysteine conjugation as described previously [31]. First, a solution of CE_3_-Ec1-(HE)_3_ or (HE)_3_-Ec1-E_3_C (0.3 mg, 16 nmoles) in degassed PBS (214 µL) was incubated with a 28-fold molar excess of DTT (70 µg, 454 nmoles, 14 µL of 5 mg/mL solution; final concentration of DTT 20 mM) at 40 °C for 30 min. The reaction mixture was purified on a NAP-5 column, pre-equilibrated with degassed 0.2 M NH_4_OAc pH 6.0. In the second step, radioiodination of ((4-hydroxyphenyl)ethyl)maleimide (HPEM) was performed. A solution of HPEM (5 µg, 23 nmoles, 5 µL of 1 mg/mL in MeOH containing 1% CH_3_COOH) was mixed with 5 µL of MeOH containing 5% CH_3_COOH, [^125^I]NaI (10–22 µL, 23–83 MBq), chloramine-T (2.5 µL of 8 mg/mL in H_2_O; 20 µg, 71 nmoles) and incubated at room temperature for 5 min. Then, sodium metabisulfite was added (2.5 µL of 12 mg/mL in H_2_O, 30 µg, 158 nmoles). The labeling yield was determined by radio-TLC analysis using silica plates on aluminum support in ethyl acetate. The radiolabeled HPEM had Rf = 0.8, and free radioiodine stayed at the application point. Immediately after purification from DTT, CE_3_-Ec1-(HE)_3_ or (HE)_3_-Ec1-E_3_C (0.27 mg, 14 nmoles, 500 µL) was added to the radiolabeled HPEM (5 µg, 23 nmoles) in a 1:1.6 molar ratio and incubated at 40 °C for 1 h. The radiolabeled Ec1 conjugates (termed [^125^I]I-HPEM-Ec1 and [^125^I]I-Ec1-HPEM, respectively) were purified using NAP-5 columns pre-equilibrated and eluted with PBS. The labeling yield was determined by radio-iTLC analysis in a 4:1 acetone:water system.

The in vitro stability test was performed by incubating [^125^I]I-HPEM-Ec1 and [^125^I]I-Ec1-HPEM with a 5000-fold molar excess of KI in PBS or with 30% ethanol at room temperature for 3 h (control samples were incubated in PBS). Samples were analyzed by iTLC in a 4:1 acetone:water system.

For labeling with cobalt-57, DOTA–Ec1 or Ec1–DOTA (10–30 µg, 0.5–1.6 nmoles in 30–70 µL 0.2 M NH_4_OAc, pH 6.5) were incubated with [^57^Co]CoCl_2_ (4–16 µL, 2–9 MBq) in 30–100 µL of 0.2 M NH_4_OAc (pH 5.5) at 60 °C for 120 min. Then, a 1000-fold molar excess of EDTA (200–600 µg, 0.5–1.6 µmoles, in 10–30 µL 0.2 M NH_4_OAc, pH 5.5) was added to the reaction mixture to complex the radiometal that was loosely bound or not chelated by the DOTA, and the reaction was incubated at 60 °C for 10 min. Purification was performed using a NAP-5 column, pre-equilibrated and eluted with PBS.

For labeling with gallium-68, DOTA–Ec1 or Ec1–DOTA (20–40 µg, 1.0–1.6 nmoles in 70–90 µL 0.2 M NH_4_OAc, pH 6.5) were incubated with [^68^Ga]GaCl_3_ (60–120 µL, 90–185 MBq) in 60–120 µL of 1.25 M NaOAc (pH 3.6) at 60 °C for 25 min. The ratio of 1:3:3 protein (µg) to [^68^Ga]GaCl_3_ eluate (µL) to NaOAc buffer (µL) was used. Then, a 1000-fold molar excess of EDTA (380–760 µg, 1.0–2.0 µmoles, in 10–20 µL of 1.25 M NaOAc, pH 3.6) was added and the reaction mixture was incubated at 60 °C for 5 min. Purification was performed using a NAP-5 column, pre-equilibrated and eluted with PBS. The values for isolated yield were corrected for decay; the reported specific activity values were at the end of purification.

For labeling with indium-111, DOTA–Ec1 or Ec1–DOTA (20–40 µg, 1.0–1.6 nmoles in 70–90 µL 0.2 M NH_4_OAc, pH 6.5) were incubated with [^111^In]InCl_3_ (50–75 µL, 42–53 MBq) in 200–250 µL of 0.2 M NH_4_OAc (pH 5.5) at 60 °C for 60 min. Then, a 1000-fold molar excess of EDTA (380–760 µg, 1.0–2.0 µmoles, in 40 µL 0.2 M NH_4_OAc, pH 5.5) was added and the reaction was incubated at 60 °C for 10 min. Purification was performed using a NAP-5 column, pre-equilibrated and eluted with PBS.

The in vitro stability test for DOTA–Ec1 or Ec1–DOTA conjugates labeled with radiometals was performed by incubating the purified proteins with a 1000-fold molar excess of EDTA in PBS (control samples were incubated in PBS). Samples were analyzed by iTLC in 0.2 M citrate buffer, pH 5.5. The values for each conjugate were normalized to its starting radiochemical purity taken as 100%.

Radio-SDS-PAGE analysis of radiolabeled compounds after purification was performed to confirm the purity and the identity. Samples of the compounds were treated with sample buffer (10 min at 70 °C) and analyzed using SDS-PAGE (200 V, 3A, 25 min) using NuPAGE 4–12% Bis-Tris Gel (Invitrogen AB, Lidingö, Sweden) in MES buffer (Invitrogen AB, Lidingö, Sweden). As markers of low-molecular-weight compounds, samples of [^57^Co]Co-EDTA, [^68^Ga]Ga-citrate, [^111^In]In-EDTA or [^125^I]NaI were applied on the same gel.

### 2.4. In Vitro Studies

In vitro studies were performed using two human prostate cancer cell lines, PC-3 and DU145, expressing EpCAM [6,9,33,34]. Evaluation of *N*- and *C*-terminal DARPin variants for each label was performed side-by-side using cells from the same passage.

The binding specificity to EpCAM in PC-3 and DU145 cells was evaluated as described previously [25]. Cells were seeded in six-well plates (VWR, Radnor, PA, USA) (ca. 5 × 10^5^ cells per well) one day before the experiment, and a set of three wells was used for each group. First, a 100-fold molar excess of non-labeled Ec1 (200 nM in 500 µL) was added to one group of cells to saturate EpCAM receptors for 30 min, while media only (500 µL) was added to the second group. Then, radiolabeled DARPin variants (4 nM in 500 µL) were added to both groups (2 nM final concentration). After incubation for 1 h at 37 °C, the supernatant was collected, cells were washed with PBS (1 mL) and 1 M NaOH (1 mL) was added to lyse the cells. The cell lysate was collected using a rubber scraper after 30 min of incubation. The activity in the supernatant and the cell-associated fractions was measured using a gamma spectrometer and the percentage of cell-bound activity was calculated.

Cellular processing of radiolabeled DARPins by DU145 cells during continuous incubation was studied by an acid-wash method [35]. Cells (ca. 7 × 10^5^ cells per dish) were seeded in 35 mm Petri dishes (Nunclon Delta Surface, ThermoFisher Scientific, Roskilde, Denmark), three dishes per time point were used. Radiolabeled DARPins (2 nM in 1 mL) were added to the cells and incubated at 37 °C in a humidified incubator for 1, 2, 3, 8 or 24 h (or for 0.5, 1, 2 and 3 h for [^68^Ga]Ga label). At the predetermined time points, the supernatant was collected, cells were washed once with PBS (1 mL) and treated with 0.2 M glycine buffer containing 4 M urea, pH 2.0 (1 mL) on ice for 5 min to collect the membrane-bound fraction. Then, the cells were washed once with the same buffer (1 mL) and were treated with 1 M NaOH (1 mL) for 30 min to collect the internalized fraction. The activity in every fraction was measured using a gamma spectrometer.

### 2.5. Measurement of Affinity Using LigandTracers

The binding kinetics of the DARPins to living DU145 cells was measured using a LigandTracer Grey for the variants radiolabeled with iodine-125 and using a LigandTracer Yellow for the variants radiolabeled with cobalt-57 or indium-111 (Ridgeview Instruments, Vänge, Sweden) as described previously [25]. Briefly, 2 × 10^6^ DU145 cells were seeded to a local area of an 89-mm Petri dish one day before the experiment. Measurements were recorded at room temperature in real-time. Two concentrations of radiolabeled DARPins (3 and 9 nM) were added to the cells during the recording of the association phase, followed by replacement of media without DARPins and measurements of retention in the dissociation phase. The dissociation constants were calculated using TraceDrawer Software (Ridgeview Instruments AB, Vänge, Sweden) based on the association and the dissociation rates.

### 2.6. Animal Studies

Animal studies were planned and performed in accordance with Swedish national legislation on the protection of laboratory animals. The studies were approved by the local ethical committee for animal research in Uppsala, Sweden (ethical permission C5/16 from 26 February 2016). 

Female BALB/c nu/nu mice (six weeks old) were supplied from Scanbur A/S (Karlslunde, Denmark) and had an adaptation period of one week before the start of experimental procedures. For implantation of xenografts, 5 × 10^6^ DU145 cells (in 100 µL of 1:1 Matrigel:media) or 5 × 10^6^ Ramos cells (in 100 µL media) were subcutaneously injected in the right hind leg of the mice. The experiments were performed three weeks after implantation. The average animal weight was 16.6 ± 1.4 g in the DU145 group, and 17.4 ± 1.3 g in the Ramos group. The average tumor weight was 0.08 ± 0.04 g for DU145 xenografts and 0.33 ± 0.14 g for Ramos xenografts. 

A dual-label approach was used to study the biodistribution of radiolabeled DARPins as described previously [25]. Four groups of mice were intravenously (i.v.) injected with a mixture of [^125^I]I-HPEM–Ec1 and [^111^In]In-DOTA–Ec1 (or [^125^I]I-Ec1–HPEM and [^111^In]In-Ec1–DOTA) in 100 μL of 1% BSA in PBS/mouse (20 KBq for [^125^I]I, 20 KBq for [^111^In]In) and dissected 3 or 24 h post injection (p.i.). Another four groups of mice were i.v. injected with a mixture of [^57^Co]Co-DOTA–Ec1 and [^68^Ga]Ga-DOTA–Ec1 (or [^57^Co]Co-Ec1–DOTA and [^68^Ga]Ga-Ec1–DOTA) in 100 μL of 1% BSA in PBS/mouse (10 KBq for [^57^Co]Co, 700 KBq for [^68^Ga]Ga-DOTA–Ec1, 1200 KBq for [^68^Ga]Ga-Ec1–DOTA) and dissected 3 or 24 h, p.i. The total injected protein amount was adjusted to 10 μg by a non-labeled Ec1. Before dissection, the mice were anesthetized by an intraperitoneal (i.p.) injection of ketamine and xylazine (250 mg/kg of ketamine, 25 mg/kg xylazine) and sacrificed by heart puncture. Blood, organs and carcass were collected and weighed, and the activity was measured using a gamma spectrometer. For samples containing iodine-125 and indium-111, the activities were measured using a protocol that allows for the detection of signals in separate energy windows for each nuclide. For samples containing gallium-68 and cobalt-57, the total radioactivity corresponding to the sum of signals was measured using an open protocol. One day after the first measurement (when gallium-68 decayed), cobalt-57 activity was measured using the same protocol. Subtraction of the cobalt-57 activity from the total signal measured on the first day was considered as gallium-68 activity. The percent of injected dose per gram of sample (%ID/g) was calculated.

Whole-body SPECT/CT scans were performed using a nanoScan SPECT/CT (Mediso Medical Imaging Systems, Budapest, Hungary). Mice bearing DU145 xenografts were injected with [^57^Co]Co-DOTA–Ec1 (10 µg, 0.9 MBq), [^57^Co]Co-Ec1–DOTA (10 µg, 0.7 MBq), [^111^In]In-DOTA–Ec1 (10 µg, 11.3 MBq), [^111^In]In-Ec1–DOTA (10 µg, 12.0 MBq), [^125^I]I-HPEM–Ec1 (10 µg, 2.0 MBq) or [^125^I]I-Ec1–HPEM (10 µg, 0.6 MBq). During imaging, the mice were anesthetized with sevoflurane. Imaging at 3 and 24 h p.i. was performed on the same mouse. The acquisition time was 20 min. CT scans were acquired using the following parameters: X-ray energy peak of 50 keV; 670 µA; 480 projections; and 5.26 min acquisition time. SPECT raw data were reconstructed using the Tera-Tomo™ 3D SPECT reconstruction technology (version 3.00.020.000; Mediso Medical Imaging Systems Ltd.): high dynamic range; 30 iterations; one subset. CT data were reconstructed using the Filter Back Projection in Nucline 2.03 Software (Mediso Medical Imaging Systems Ltd.). SPECT and CT files were fused using the Nucline 2.03 Software and are presented as maximum intensity projections in an RGB color scale.

Whole-body PET/CT scans were acquired using a nanoScan PET/MRI scanner (Mediso Medical Imaging Systems Ltd., Budapest, Hungary). Mice bearing DU145 xenografts were injected with [^68^Ga]Ga-DOTA–Ec1 (10 µg, 6.9 MBq) or [^68^Ga]Ga-Ec1–DOTA (10 µg, 5.9 MBq) and imaged 3 h p.i. Immediately before imaging, the mice were sacrificed by CO_2_ asphyxiation. PET scans were performed for 60 min followed by CT scan. CT was acquired using the same bed position as PET at the following parameters: CT energy peak of 50 keV, 670 μA, 480 projections and 5.26 min acquisition time. The PET data were reconstructed using the Tera-Tomo™ 3D reconstruction engine. The CT data were reconstructed using the Filter Back Projection in Nucline 2.03 Software (Mediso Medical Imaging Systems, Budapest, Hungary). The PET and the CT files were fused using Nucline 2.03 Software and analyzed using the PMOD 4.002 software (PMOD Technologies, Zürich, Switzerland).

## 3. Results

### 3.1. Protein Production, Purification and Conjugation

Two Ec1 variants, having a cysteine either at the N-terminus (CE_3_-Ec1-(HE)_3_) or at the *C*-terminus ((HE)_3_-Ec1-E_3_C) were produced in E. coli, purified and conjugated to a maleimide-DOTA chelator (DOTA–Ec1 and Ec1–DOTA). The molecular weights of the proteins after conjugation were confirmed by mass spectrometry (Supplementary Figure S1). The obtained values for the other variants matched the theoretical values within 1 Da accuracy.

### 3.2. Radiolabeling and Stability

DOTA-conjugated Ec1 variants were labeled with cobalt-57, gallium-68 or indium-111 and were purified using NAP-5 size-exclusion columns providing radiochemical purities over 96% (Table 1). For indirect radioiodination, a bifunctional maleimide-containing HPEM linker was iodinated first and then [^125^I]I-HPEM was conjugated to CE_3_-Ec1-(HE)_3_ and (HE)_3_-Ec1-E_3_C. The radioiodinated conjugates were purified using NAP-5 columns, providing radiochemical purities over 98%.

SDS-PAGE analysis of the labeled Ec1 variants after purification showed a single peak for Ec1 variants labeled with cobalt-57, indium-111 and iodine-125 (Supplementary Figures S2 and S3). These variants also demonstrated high stability during incubation with a 1000-fold molar excess of EDTA for up to 3 h (Table 2 and Table 3). Ec1 variants labeled with gallium-68 demonstrated 4–5% release of activity after 1 h and 8–10% after 3 h of incubation in PBS; the same level of release was observed during incubation with EDTA. Some release of activity (12%) was also observed during SDS-PAGE analysis of these variants (Supplementary Figure S3).

### 3.3. In Vitro Studies

**Figure 2:**
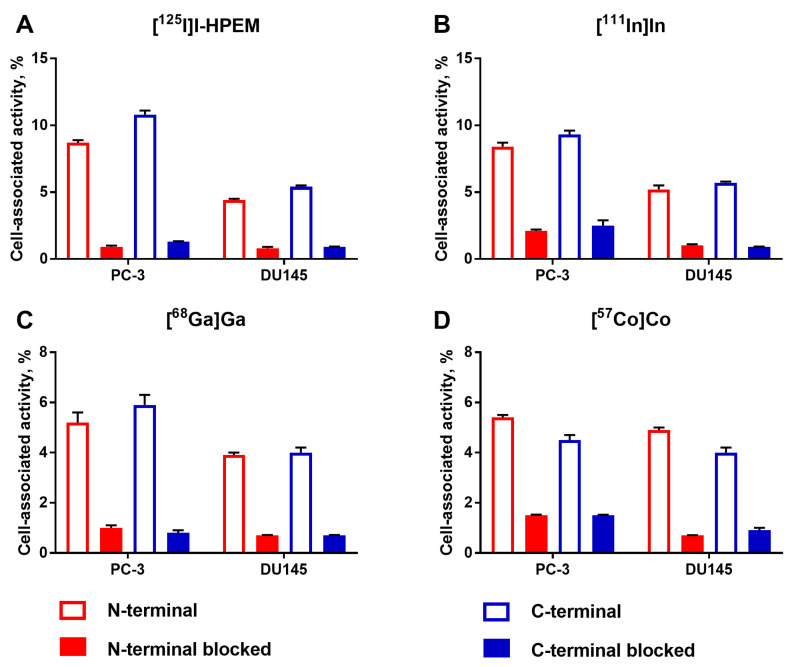
Binding specificity of Ec1 conjugates radiolabeled with (**A**) [^125^I]I-HPEM, (**B**) [^111^In]In, (**C**) [^68^Ga]Ga and (**D**) [^57^Co]Co at the *N*- or the *C*-terminus, to EpCAM-expressing PC-3 and DU145 prostate cancer cells in vitro. For blocking, a 100-fold molar excess of non-labeled Ec1 was added to the blocked groups. The final concentration of radiolabeled compounds was 2 nM. The data are presented as mean from three samples ± SD.

Binding specificity of radiolabeled Ec1 variants to EpCAM-expressing PC-3 and DU145 prostate cancer cells was studied by saturating the EpCAM receptors with a large excess of unlabeled Ec1. A significant (*p* < 0.05) reduction in cell-associated activity was observed in the blocked groups, which confirmed specific binding of all Ec1 variants (Figure 2).

Measurements of the binding kinetics of Ec1 variants to DU145 cells showed that the variants labeled with indium-111 and iodine-125 had similar equilibrium dissociation constants (K*_D_* values) between 0.20 and 0.36 nM, while the variants labeled with cobalt-57 had somewhat higher K*_D_* values (0.60 ± 0.25 and 0.66 ± 0.32 nM, for *N*- and *C*-terminus, respectively) (Table 4). No major differences between the variants with *N*- or *C*-terminal position of the label were observed.

The results of cellular processing showed that the internalization rate of Ec1 variants in DU145 cells was low (Supplementary Figures S4 and S5). The Ec1 variants labeled with indium-111 or cobalt-57 had less than 5% of total cell-associated activity internalized by 3 h and 15–20% by 24 h. The Ec1 variants labeled with gallium-68 had a slightly higher internalization rate with ca. 10% internalized by 3 h. The patterns of cellular processing for the variants with *N*- or *C*-terminal label positions were similar.

### 3.4. In Vivo Studies

Biodistribution comparison of radiolabeled Ec1 variants was performed in BALB/c nu/nu mice bearing DU145 xenografts 3 h post injection (p.i.) for all eight variants (Table 5) and 24 h p.i. for the variants labeled with cobalt-57, indium-111 and iodine-125 (Table 6).

**Figure 3:**
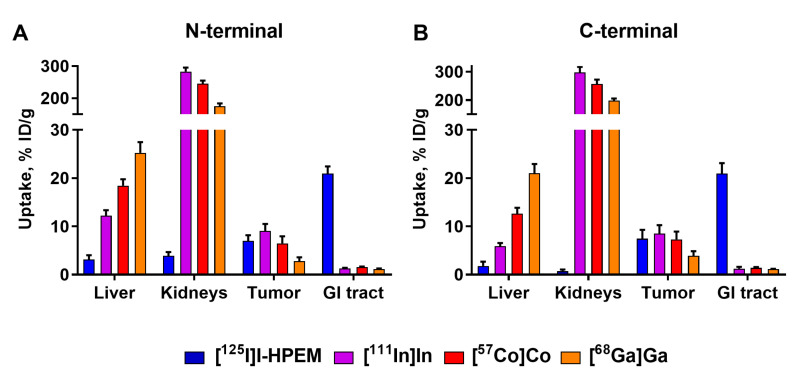
Influence of position ((**A**) N-terminal or (**B**) *C*-terminal) and composition ([^125^I]I-HPEM, [^111^In]In, [^68^Ga]Ga, [^57^Co]Co) of the label on biodistribution of DARPin Ec1 variants 3 h post injection (p.i.) in Balb/c nu/nu mice bearing DU145 prostate cancer xenografts. Uptake is presented as % ID/g (average from four mice ± SD; from seven mice ± SD for the [^57^Co]Co *C*-terminal group). Uptake in gastrointestinal (GI) tract with content is presented as % ID per whole sample.

Overall, the biodistribution of radiometal-labeled Ec1 variants was typical for DARPins, with fast clearance from blood via kidneys followed by renal reabsorption and high retention of activity in kidneys. The analysis of the biodistribution data demonstrated a profound influence of the label type and its position on the uptake in normal organs (Figure 3). Among the radiometals, [^111^In]In-labeled variants had the lowest uptake in liver, followed by the variants labeled with cobalt-57 and gallium-68. The increase in liver uptake correlated with the decrease in uptake in kidneys. Overall, all variants with a *C*-terminal location of the label had lower uptake in normal organs than the variants with an N-terminal position, including the [^125^I]I-HPEM label. The activity uptake in blood at 3 h p.i. was below or equal to 1% ID/g, being the lowest for the *C*-terminal [^111^In]In and [^57^Co]Co labels. The tumor uptake of *C*-terminal [^111^In]In-Ec1–DOTA (9 ± 2% ID/g) was similar to [^57^Co]Co-Ec1–DOTA (7 ± 2% ID/g). All Ec1 variants labeled with radiometals at the *C*-terminus had a similar uptake in muscles and bone. Accordingly, at 3 h p.i., Ec1 variants with *C*-terminal indium-111 or cobalt-57 labels provided similar tumor-to-blood, tumor-to-liver, tumor-to-lung and tumor-to-muscle ratios (*p* > 0.05, one-way ANOVA test), with a significantly (*p* < 0.05, one-way ANOVA test) higher tumor-to-bone ratio for indium-111 (Table 7). 

**Figure 4:**
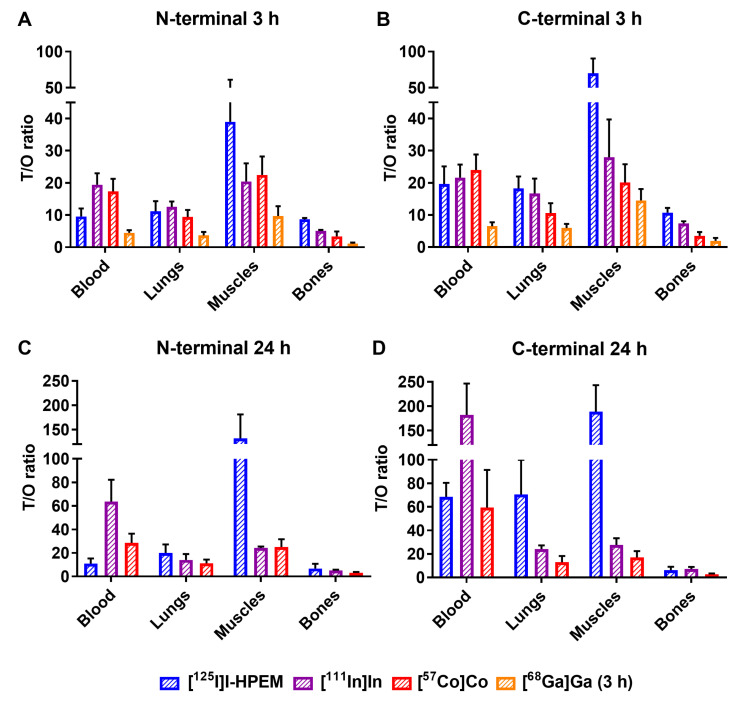
Tumor-to-normal-tissue ratios of DARPin Ec1 variants radiolabeled with [^125^I]I-HPEM, [^111^In]In, [^57^Co]Co and [^68^Ga]Ga at *N*- (**A,C**) or *C*-terminus (**B,D**) in Balb/c nu/nu mice bearing EpCAM-expressing DU145 xenografts at 3 or 24 h p.i.

Ec1 variants labeled with gallium-68 had ca. two-fold lower tumor uptake, which was at the same level as the uptake in bones 3 h p.i. The activity uptake in blood was ca. 1.5-fold higher than for the other radiometals. In combination with a high uptake in normal organs (liver, spleen), this resulted in the lowest tumor-to-organ ratios for gallium-68 among the studied radiolabels (Figure 4).

Ec1 variants labeled with [^125^I]I-HPEM at the *C*-terminus had a significantly (*p* < 0.05, one-way ANOVA test) lower retention of activity in lungs, liver, spleen, kidneys, muscles and bones than the radiometal labels, indicating a non-residualizing character of the [^125^I]I-HPEM label. The tumor uptake of [^125^I]I-HPEM-Ec1 variants was as high as the variants labeled with indium-111 and cobalt-57. However, the majority of [^125^I]I activity at 3 h p.i. was detected in the gastrointestinal tract (21 ± 2% ID), which suggested excretion via the hepatobiliary system.

By 24 h p.i., the advantage of the *C*-terminal over the N-terminal position of the label became more pronounced for all variants, with the increase in tumor-to-blood ratio for the *C*-terminal labels (Table 6 and Table 8). Although both [^111^In]In-Ec1–DOTA and [^57^Co]Co-Ec1–DOTA had good retention in tumors and provided similar tumor-to-organ ratios (*p* > 0.05 for all, except tumor-to-blood; one-way ANOVA test), there was a tendency for higher tumor-to-organ ratios for the indium-111 label.

**Figure 5:**
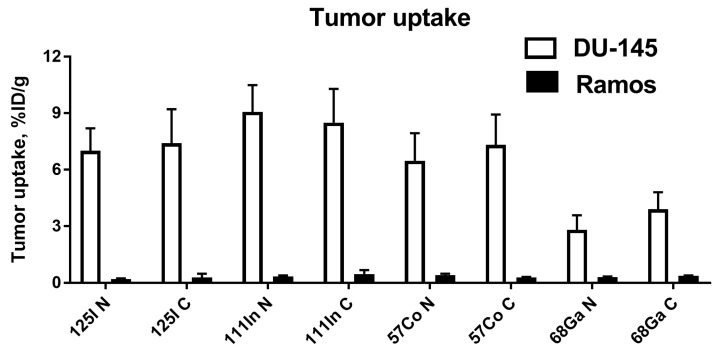
Tumor-targeting specificity of DARPin Ec1 variants labeled with [^57^Co]Co, [^68^Ga]Ga, [^111^In]In and [^125^I]I-HPEM at *N*- or *C*-terminus 3 h post injection (p.i.) in Balb/c nu/nu mice bearing EpCAM-expressing DU145 xenografts (*n* = 4–7) and EpCAM-negative Ramos xenografts (control, *n* = 4). Uptake is presented as % ID/g ± SD.

The specificity of EpCAM targeting by the radiolabeled Ec1 variants was confirmed using BALB/c nu/nu mice bearing Ramos xenografts 3 h p.i. (Figure 5). The tumor uptake of all variants in Ramos xenografts was significantly (*p* < 0.05, unpaired *t*-test) lower compared with the uptake in DU145 xenografts.

**Figure 6:**
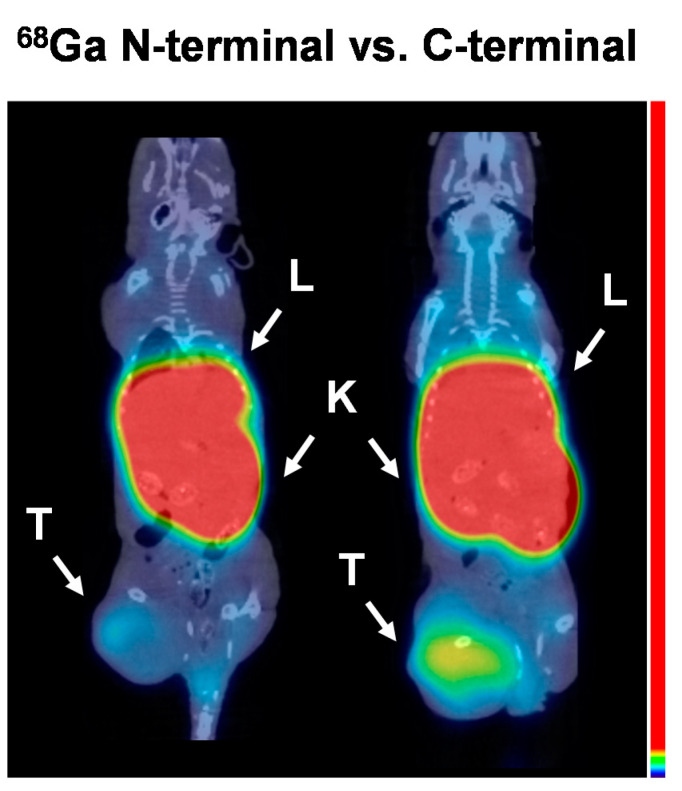
Positron Emission Tomography/Computed Tomography (microPET/CT) imaging of EpCAM expression in Balb/c nu/nu mice bearing EpCAM-expressing DU145 xenografts at 3 h post injection (p.i.) of DARPin Ec1 variants labeled with [^68^Ga]Ga at *N*- or *C*-terminus. Arrows indicate: T—tumor, K—kidneys, L—liver. The same color scale (adjusted to the first red pixel in the tumor in the right image) was applied for both imaging. Lower SUV threshold 0.0, upper SUV threshold 1.0.

**Figure 7:**
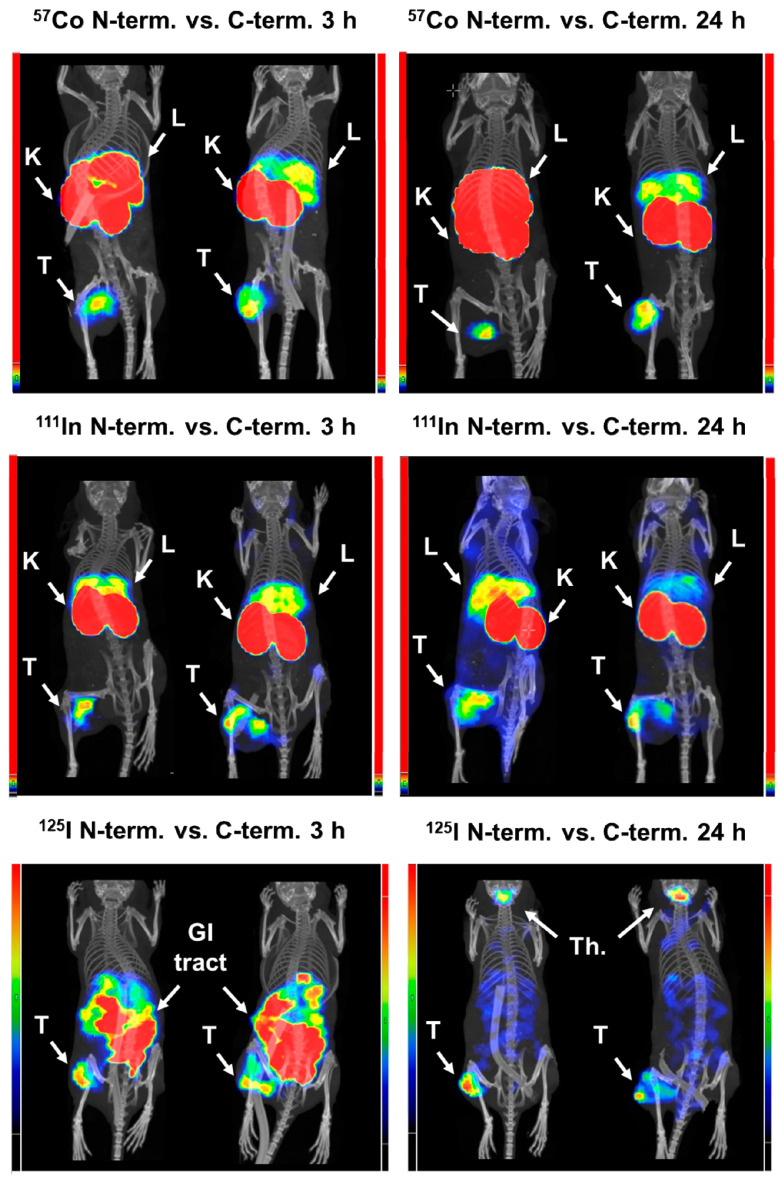
Micro-Single-Photon Emission Computed Tomography/Computed Tomography (microSPECT/CT) imaging of EpCAM expression in Balb/c nu/nu mice bearing EpCAM-expressing DU145 xenografts at 3 and 24 h post injection (p.i.). Ec1 variants labeled with [^57^Co]Co, [^111^In]In and [^125^I]I-HPEM at *N*- or *C*-terminus. Arrows indicate: T—tumor, K—kidneys, L—liver, Th.—thyroid. The relative color scales are adjusted to the first red pixel in the tumor. Left scales are related to imaging using variants with a label on the N-terminus and right scales to imaging using variants with a label on the *C*-terminus.

The biodistribution data were confirmed by experimental microPET/CT imaging for Ec1 variants labeled with gallium-68 3 h p.i (Figure 6) and by microSPECT/CT imaging for Ec1 variants labeled with cobalt-57, indium-111 and iodine-125, 3 and 24 h p.i. (Figure 7).

## 4. Discussion

The application of radiolabeled monoclonal antibodies and their fragments and derivatives as imaging probes is based on several decades of intensive preclinical investigations and clinical applications [20]. The investigation of DARPins for radionuclide imaging purposes is much newer. Still, preclinical studies [28,31] and results of a Phase I clinical trial [36] suggest that DARPins can image HER2-expressing tumors within 2–4 h after injection and identify patients for HER2-targeting therapies. Our recent work shows that Ec1 derivatives might serve the same purpose for EpCAM targeting [23,24,25]. Still, a lot more knowledge concerning the structure–property relationship is required to reveal the full potential of DARPins as imaging probes and to enable a tailored design for specific applications. This study belongs to a series of investigations aiming to elucidate the impact of labeling chemistry on the imaging properties of DARPins. 

The major rationale for the study design is that the charge and geometry of the chelator–metal complex as well as local hydrophilicity might affect the interaction of the tracer with blood proteins and scavenger receptors in normal tissues (e.g., the liver). It has to be noted that these physicochemical features also depend on the characteristics of adjacent amino acids. Accordingly, one can expect that the same combination of a nuclide and a chelator would have a different impact depending on its position on the ESP. This was clearly shown for imaging probes based on another class of ESPs, the affibody molecules [27]. Similar observations were made for probes based on yet another ESP scaffold, the ADAPTs (Albumin-binging domain-Derived Affinity ProTeins) [37,38]. The present study also demonstrated a strong impact of the label position on the distribution of labeled Ec1 (Figure 3, Table 5 and Table 6). Positioning of a label at the *C*-terminus resulted in lower uptake in normal tissues. This translated into high tumor-to-organ ratios for this type of conjugates, as the position of a label had no impact on the tumor uptake of conjugates with the same label (Figure 5).

The imaging properties of ^68^Ga-labeled Ec1 variants were disappointing, independent of the label position (Figure 5 and Figure 6). These probes had a twice lower uptake in tumors at 3 h p.i. compared to other radiometals. The tumor uptake was at the same level as uptake in bones, making these tracers suboptimal for PCa imaging when high imaging contrast in bones is required, as in the case of oligometastatic prostate cancer. Low tumor uptake was likely due to a combination of instability of ^68^Ga-label observed during the in vitro tests (Table 2) and high liver uptake (Figure 3), which could have decreased the bioavailability of [^68^Ga]Ga-labeled tracers. Although the thermodynamic stability of the DOTA–gallium complex (log K_DOTA-Ga(III)_ = 21.3) is lower than the stability of Ga complexes with other chelators, e.g., NOTA (log K_NOTA-Ga(III)_ = 31.0) [39], DOTA is commonly used for gallium-68 labeling as it often provides favorable biodistribution [30,40]. A possible explanation for the insufficient stability of the ^68^Ga label in our case is the relatively low temperature of labeling (60 °C). This might lead to the formation of out-of-cage complexes with higher lability [41]. However, increasing the labeling temperature from 60 to 85 °C resulted in a significant loss of Ec1 binding to EpCAM-expressing cells. Apparently, DOTA is a suboptimal chelator for labeling of Ec1 with ^68^Ga. Future studies should be focused on other chelators, such as NOTA or NODAGA, which provide better stability at a lower labeling temperature.

Positioning the indium-111 and cobalt-57 labels at the *C*-terminus resulted in conjugates which performed nearly equally well at 3 and at 24 h (Table 5 and Table 6). Although there were no statistically significant differences in the majority of organs and tissues (except bone), there was a tendency for higher tumor-to-organ ratios for indium-111 (Table 7 and Table 8). ^111^In label also provided the highest tumor-to-blood ratio in this study (182 ± 64 at 24 h after injection). A critically important result is that the tumor-to-bone ratio for ^111^In was twice higher compared to ^57^Co. This feature makes ^111^In the preferred label, as prostate cancer is prone to formation of bone metastases. It has to be noted that the use of ^111^In for SPECT imaging is not the only opportunity for an application of indium radioisotopes for labeling of proteins and peptides. For PET imaging, ^110m^In could be used. This radionuclide emits positrons with a branching ratio of 61.3% and has a half-life of 69 min, i.e. close to the half-life of ^68^Ga [42]. This radionuclide can be produced with a high yield using low-energy cyclotrons, which are widely available at PET centers [43]. A pilot clinical study demonstrated that ^110m^In can be used for labeling of tumor-targeting peptides and provides high-resolution imaging [44]. The half-life of ^110m^In is compatible with imaging at 3 h after injection, when In-labeled Ec1 provides a good contrast (Table 7).

Somewhat disappointing is the high uptake and persistent retention of activity in kidneys in the case of DARPins with residualizing radiometal labels (Table 5 and Table 6; Figure 6 and Figure 7). This finding is consistent with our previous data for anti-HER2 [28,32] and anti-EpCAM [23] DARPins with residualizing ^99m^Tc label. Autoradiography studies [45] have shown that the activity is accumulated in the renal cortex, presumably in proximal tubuli. Common methods of prevention of the renal re-absorption of radiolabeled peptides, such as co-injection of Gelofusine or lysine, did not reduce renal uptake of radiolabeled DARPins [45]. Inefficient also were pre-injections of colchicine, probenecid, mannitol or furosemide. It has to be noted that renal metastases of prostate cancer are encounter quite rarely, in less than 1% of patients [46]. One could expect that they will be even rarer at the oligometastatic stage. Therefore, the high renal uptake should not appreciably affect the accuracy of EpCAM imaging in oligometastatic prostate cancer using radiometal-labeled Ec1.

The tumor uptake of ^125^I-labeled variants at 3 h p.i. was at the same level as for indium-111 and cobalt-57 (Figure 5). This is in agreement with the in vitro data demonstrating slow internalization of Ec1 conjugates (Supplementary Figures S5 and S6). In the case of slow internalization, the cellular retention of the non-residualizing radioiodine label was as good as the retention of the radiometals, because the majority of activity was membrane-bound. However, retention of radioiodine in the tumors was significantly lower at 24 h compared to the radiometals. This could be explained by internalization, lysosomal degradation of the proteins and release of radiocatabolites. 

The retention of iodine-labeled Ec1 in normal tissues was low (Figure 3; Table 5 and Table 6). This can be attributed to a different mechanism and rate of internalization than is observed in the tumors. Apparently, the internalization in normal tissues is caused by off-target interactions and is quicker than in tumors. Therefore, the release of radiometabolites is substantial already 3 h after injection. Overall, a slow release of radiometabolites from the tumors but rapid release from normal tissues was favorable for achieving the high tumor-to-organ ratios. One of the downsides of this phenomenon was a noticeable accumulation of radioiodide, the ultimate radiometabolite of ^125^I-HPEM, in thyroid 24 h after injection (Figure 7). This accumulation was equally prominent for positioning of the label at both the C- and N-terminus of the DARPin. It has to be noted that the elevated thyroid uptake should not compromise the imaging of oligometastatic prostate cancer, when both the primary tumor and metastases are located in pelvis. 

A prominent feature of radioiodinated Ec1 was appreciable hepatobiliary excretion. The activity in the content of the gastrointestinal tract was 21 ± 1% of the injected activity, independent of label position. Such a pattern was also observed earlier for the anti-HER2 DARPin G3, labeled using [^125^I]I-HPEM [31]. Usually, the hepatobiliary excretion is considered undesirable as it creates a high background in the peritoneum and complicates the imaging of abdominal metastases. However, this can be an advantage for imaging of oligometastatic prostate cancer. In this case, both primary tumor and metastases are located close to the urinary bladder. A high activity in urine complicates imaging, and prostate cancer patients experience a problem with complete voiding of the bladder. Switching to hepatobiliary excretion enables reduction in activity in the urinary bladder, and therefore minimizes the background in this case. Overall, the use of the radioiodine label in combination with the HPEM linker at the *C*-terminus was the most favorable for visualization of EpCAM expression in prostate cancer.

## 5. Conclusions

In conclusion, the position, composition and physicochemical properties of the label are important to achieve the maximum contrast of imaging of EpCAM expression using the DARPin Ec1. The *C*-terminus is a preferable position for any of the radioactive labels. The non-residualizing [^125^I]I-HPEM label provided the highest tumor-to-muscle and tumor-to-bone ratios 3 h p.i. and is the most suitable for imaging of EpCAM in the early stages of prostate cancer. Among the radiometals, indium-111 at the *C*-terminus provided as high a tumor-to-blood ratio as [^125^I]I-HPEM, the highest tumor-to-lung and tumor-to-liver ratios and could be used in late-stage prostate cancer.

## Figures and Tables

**Table 1:** Results of radiolabeling of Ec1 variants.

Compound	Radiochemical Yield (Non-Isolated), %	Radiochemical Yield (Isolated), %	Radiochemical Purity, %	Maximal Specific Activity, Mbq/µg (Mbq/Nmol)
**[^125^I]I-HPEM (*n* = 2)**	86 ± 10	n/a	n/a	13.8 (3.0)
**[^125^I]I-HPEM–Ec1 (*n* = 2)**	53 ± 1	39 ± 0	99 ± 1	0.18 (3.3)
**[^125^I]I-Ec1–HPEM (*n* = 2)**	19 ± 4	16 ± 4	98 ± 0	0.05 (0.9)
**[^57^Co]Co-DOTA–Ec1 (*n* = 1)**	34	26	97	0.1 (1.9)
**[^57^Co]Co-Ec1–DOTA (*n* = 2)**	62 ± 16	35 ± 26	99 ± 1	0.08 (1.4)
**[^68^Ga]Ga-DOTA–Ec1 (*n* = 4)**	62 ± 6	29 ± 3	97 ± 1	2.0 (38.4)
**[^68^Ga]Ga-Ec1–DOTA (*n* = 3)**	71 ± 8	36 ± 7	98 ± 0	1.8 (34.6)
**[^111^In]In-DOTA–Ec1 (*n* = 2)**	82 ± 3	66 ± 2	98 ± 1	1.8 (34.6)
**[^111^In]In-Ec1–DOTA (*n* = 2)**	84 ± 2	67 ± 3	98 ± 0	1.7 (33.2)

**Table 2:** In vitro stability of Ec1 conjugates labeled with radiometals. The conjugates were incubated for 1 and 3 h with a 1000-fold molar excess of EDTA and compared to PBS control. Analysis was performed in duplicates. The values for each conjugate were normalized to its starting radiochemical purity, taken as 100%.

	Protein-Associated Activity, %			
	1 h		3 h	
	PBS	1000× EDTA	PBS	1000× EDTA
**[^57^Co]Co-DOTA–Ec1**	100 ± 0	100 ± 0	100 ± 0	100 ± 0
**[^57^Co]Co-Ec1–DOTA**	100 ± 0	99 ± 0	100 ± 0	99 ± 0
**[^68^Ga]Ga-DOTA–Ec1**	96 ± 0	95 ± 0	93 ± 0	92 ± 0
**[^68^Ga]Ga-Ec1–DOTA**	95 ± 0	95 ± 0	91 ± 1	93 ± 1
**[^111^In]In-DOTA–Ec1**	99 ± 0	99 ± 0	100 ± 0	99 ± 0
**[^111^In]In-Ec1–DOTA**	99 ± 0	99 ± 0	99 ± 0	99 ± 0

**Table 3:** In vitro stability of radioiodinated Ec1 conjugates. The conjugates were incubated for 3 h with 30% ethanol or with a 5000-fold molar excess of KI in PBS and compared to PBS control. Analysis was performed in duplicates. The values for each conjugate were normalized to its starting radiochemical purity, taken as 100%.

	Protein-Associated Activity, %		
	PBS	30% EtOH	5000× KI
**[^125^I]I-HPEM–Ec1**	100 ± 0	100 ± 0	100 ± 0
**[^125^I]I-Ec1–HPEM**	99 ± 1	100 ± 0	100 ± 0

**Table 4:** Dissociation equilibrium constants (K*_D_*) for the interaction between Ec1 variants labeled with [^57^Co]Co, [^111^In]In and [^125^I]I-HPEM at *N*- or *C*-terminus, and DU145 prostate cancer cells.

Compound/Label Position	K*_D_*, nM	
	*N*-Terminal	*C*-Terminal
**[^57^Co]Co-Ec1 (*n* = 2)**	0.60 ± 0.25	0.66 ± 0.32
**[^111^In]In-Ec1 (*n* = 2)**	0.28 ± 0.06	0.21 ± 0.01
**[^125^I]I-HPEM-Ec1 (*n* = 2)**	0.27 ± 0.03	0.32 ± 0.04

**Table 5:** Comparative biodistribution of DARPin Ec1 variants labeled with [^57^Co]Co, [^68^Ga]Ga, [^111^In]In and [^125^I]I-HPEM at *N*- or *C*-terminus 3 h post injection (p.i.) in Balb/c nu/nu mice bearing EpCAM-expressing DU145 xenografts. Uptake is presented as % injected dose (ID)/g (average from 4 mice ± standard deviation (SD); from 7 mice ± SD for the [^57^Co]Co C group). Data for intestines with content and carcass are presented as % ID per whole sample. One-way ANOVA with Bonferroni’s multiple comparisons test was performed to find significant differences between Ec1 variants with the same label position (*N*- or *C*-terminus).

	[^57^Co]Co N	[^57^Co]Co C	[^68^Ga]Ga N	[^68^Ga]Ga C	[^111^In]In N	[^111^In]In C	[^125^I]I N	[^125^I]I C
**Blood**	0.4 ± 0.1 ^a,c,g^	0.3 ± 0.1 ^a,b^	0.6 ± 0.1	0.6 ± 0.1 ^d,e^	0.47 ± 0.03 ^f,g^	0.4 ± 0.1	0.8 ± 0.2 ^g^	0.4 ± 0.1
**Sal. gland**	1.0 ± 0.2	1.2 ± 0.2	1.0 ± 0.1	0.8 ± 0.1	1.3 ± 0.2	1.0 ± 0.2	2 ± 1	1.4 ± 0.8
**Lung**	0.7 ± 0.1	0.6 ± 0.1 ^c^	0.77 ± 0.03	0.7 ± 0.1 ^e^	0.7 ± 0.1 ^g^	0.5 ± 0.1	0.7 ± 0.1 ^g^	0.40 ± 0.04
**Liver**	18 ± 1 ^a,b,c,g^	13 ± 1 ^a,b,c^	25 ± 2 ^d,e,g^	21 ± 2 ^d,e^	12 ± 1 ^f,g^	6 ± 1 ^f^	3 ± 1	2 ± 1
**Spleen**	3.2 ± 0.5 ^a,c,g^	2.6 ± 0.3 ^a, c^	6 ± 1 ^d,e^	5 ± 1 ^d,e^	2.4 ± 0.3 ^f,g^	1.4 ± 0.3 ^f^	0.5 ± 0.1	0.3 ± 0.1
**Stomach**	0.8 ± 0.1 ^c^	0.9 ± 0.1	0.9 ± 0.2 ^e^	0.69 ± 0.03	1.0 ± 0.2 ^f^	0.7 ± 0.1	1.9 ± 0.4	1.2 ± 0.6
**Kidney**	246 ± 9 ^a,b,c^	257 ± 15 ^a,b,c^	175 ± 9 ^d,e,g^	198 ± 8 ^d,e^	283 ± 12 ^f^	298 ± 20 ^f^	4 ± 1 ^g^	0.7 ± 0.4
**Tumor**	7 ± 2 ^a^	7 ± 2 ^a^	3 ± 1 ^d,e^	4 ± 1 ^d,e^	9 ± 1	9 ± 2	7 ± 1	7 ± 2
**Muscle**	0.3 ± 0.1	0.3 ± 0.1 ^c^	0.3 ± 0.1	0.27 ± 0.04	0.5 ± 0.1 ^f^	0.3 ± 0.1 ^f^	0.2 ± 0.1	0.11 ± 0.07
**Bone**	2 ± 1 ^c^	2 ± 1 ^c^	3 ± 1 ^e^	2 ± 1 ^d,e^	1.8 ± 0.4 ^g^	1.2 ± 0.3	0.8 ± 0.1	0.7 ± 0.2
**Intestines**	1.6 ± 0.1 ^c^	1.4 ± 0.2 ^c^	1.2 ± 0.1 ^e^	1.1 ± 0.1	1.3 ± 0.1 ^f^	1.2 ± 0.4 ^f^	21 ± 2	21 ± 2
**Carcass**	8 ± 1 ^c^	9 ± 1 ^c^	8 ± 1 ^e^	8 ± 1 ^e^	10 ± 1 ^f^	10 ± 1 ^f^	4 ± 1	3 ± 1

^a^ Significant difference (*p* < 0.05) between [^57^Co]Co and [^68^Ga]Ga. ^b^ Significant difference (*p* < 0.05) between [^57^Co]Co and [^11^1In]In. ^c^ Significant difference (*p* < 0.05) between [^57^Co]Co and [^125^I]I. ^d^ Significant difference (*p* < 0.05) between [^68^Ga]Ga and [^111^In]In. ^e^ Significant difference (*p* < 0.05) between [^68^Ga]Ga and [^125^I]I. ^f^ Significant difference (*p* < 0.05) between [^111^In]In and [^125^I]I. ^g^ Significant difference (*p* < 0.05) between *N*- and *C*-terminal placement of the label (unpaired *t*-test).

**Table 6:** Comparative biodistribution of DARPin Ec1 variants labeled with [^57^Co]Co, [^111^In]In or [^125^I]I-HPEM at *N*- or *C*-terminus 24 h p.i. in Balb/c nu/nu mice bearing EpCAM-expressing DU145 xenografts. Uptake is presented as % ID/g (average from 4 mice ± SD). Data for intestines with content and carcass are presented as % ID per whole sample. One-way ANOVA with Bonferroni’s multiple comparisons test was performed to find significant differences between Ec1 variants with the same label position (*N*- or *C*-terminus).

	[^57^Co]Co N	[^57^Co]Co C	[^111^In]In N	[^111^In]In C	[^125^I]I N	[^125^I]I C
**Blood**	0.15 ± 0.02 ^a,d^	0.07 ± 0.02 ^a^	0.08 ± 0.02 ^c,d^	0.04 ± 0.01	0.15 ± 0.01 ^d^	0.046 ± 0.003
**Salivary gland**	0.7 ± 0.1 ^a,b^	0.9 ± 0.2 ^b^	1.0 ± 0.1 ^c^	0.9 ± 0.2 ^c^	0.05 ± 0.02 ^d^	0.020 ± 0.004
**Lung**	0.40 ± 0.04 ^b,d^	0.31 ± 0.02 ^b^	0.4 ± 0.1 ^c,d^	0.30 ± 0.03 ^c^	0.08 ± 0.01 ^d^	0.05 ± 0.01
**Liver**	12 ± 1 ^a,b,d^	9 ± 1 ^a,b^	8 ± 1 ^c,d^	5 ± 1 ^c^	0.3 ± 0.1 ^d^	0.21 ± 0.01
**Spleen**	2.6 ± 0.4 ^b^	3 ± 1 ^a,b^	3 ± 1 ^c,d^	1.4 ± 0.1 ^c^	0.09 ± 0.02	0.08 ± 0.02
**Stomach**	0.5 ± 0.1 ^b^	0.6 ± 0.1 ^b^	0.7 ± 0.1 ^c^	0.5 ± 0.1 ^c^	0.05 ± 0.03	0.06 ± 0.02
**Kidney**	197 ± 15 ^b^	185 ± 12 ^a,b^	204 ± 15 ^c^	231 ± 19 ^c^	0.7 ± 0.3 ^d^	0.2 ± 0.1
**Tumor**	4 ± 2	4 ± 2 ^a^	5 ± 2 ^c^	7 ± 2 ^c^	2 ± 1 ^d^	3 ± 1
**Muscle**	0.17 ± 0.02 ^b^	0.20 ± 0.03 ^b^	0.3 ± 0.1 ^c^	0.3 ± 0.1 ^c^	0.02 ± 0.01	0.02 ± 0.01
**Bone**	1.5 ± 0.4 ^b^	1.3 ± 0.4 ^b^	1.3 ± 0.2 ^c^	1.1 ± 0.3	0.4 ± 0.2	0.6 ± 0.2
**Intestines**	1.0 ± 0.1	1.0 ± 0.2 ^a,b^	1.0 ± 0.2 ^c,d^	0.8 ± 0.1 ^c^	0.2 ± 0.1	0.2 ± 0.1
**Carcass**	5.3 ± 0.3 ^a,b,d^	6 ± 1 ^b^	7 ± 1 ^c^	6 ± 1 ^c^	0.9 ± 0.2	1.0 ± 0.3

^a^ Significant difference (*p* < 0.05) between [^57^Co]Co and [^111^In]In. ^b^ Significant difference (*p* < 0.05) between [^57^Co]Co and [^125^I]I. ^c^ Significant difference (*p* < 0.05) between [^111^In]In and [^125^I]I. ^d^ Significant difference between *N*- and *C*-terminal placement of the label (unpaired *t*-test).

**Table 7:** Tumor-to-normal-tissue ratios for DARPin Ec1 variants labeled with [^57^Co]Co, [^68^Ga]Ga, [^111^In]In and [^125^I]I-HPEM at *N*- or *C*-terminus 3 h p.i. in Balb/c nu/nu mice bearing EpCAM-expressing DU145 xenografts (average from 4 mice ± SD; from 7 mice ± SD for the [^57^Co]Co C group). One-way ANOVA with Bonferroni’s multiple comparisons test was performed to find significant differences between Ec1 variants with the same label position (*N*- or *C*-terminus).

	[^57^Co]Co N	[^57^Co]Co C	[^68^Ga]Ga N	[^68^Ga]Ga C	[^111^In]In N	[^111^In]In C	[^125^I]I N	[^125^I]I C
**Blood**	17 ± 4 ^a,c,g^	24 ± 5 ^a^	5 ± 1 ^d,g^	7 ± 1 ^d,e^	20 ± 4 ^f^	22 ± 4	10 ± 3 ^g^	20 ± 6
**Sal. gland**	7 ± 2 ^a^	6 ± 2	3 ± 1 ^d,g^	5 ± 1	8 ± 2 ^f^	9 ± 3	4 ± 2	6 ± 3
**Lung**	9 ± 2 ^a^	11 ± 3 ^c^	4 ± 1 ^d,e,g^	6 ± 1 ^d,e^	13 ± 2	17 ± 5	11 ± 3 ^g^	18 ± 4
**Liver**	0.4 ± 0.1 ^c^	0.5 ± 0.2 ^c^	0.11 ± 0.02 ^e,g^	0.18 ± 0.03 ^e^	0.7 ± 0.1 ^f,g^	1.5 ± 0.4 ^f^	2.4 ± 0.8	5 ± 3
**Spleen**	2 ± 1 ^c^	3 ± 1 ^c^	0.5 ± 0.1 ^e,g^	0.9 ± 0.3 ^e^	4 ± 1 ^f,g^	6 ± 1 ^f^	16 ± 6	24 ± 6
**Stomach**	8 ± 1 ^a,c^	8 ± 2	3 ± 1 ^d,g^	6 ± 1 ^d^	9 ± 1 ^f^	11 ± 4	4 ± 1	7 ± 3
**Kidney**	0.03 ± 0.01 ^c^	0.03 ± 0.01 ^c^	0.016 ± 0.004 ^e^	0.02 ± 0.01 ^e^	0.03 ± 0.01 ^f^	0.03 ± 0.01 ^f^	1.8 ± 0.6 ^g^	13 ± 9
**Muscle**	22 ± 6	20 ± 6 ^c^	10 ± 3 ^e^	15 ± 4 ^e^	20 ± 6	28 ± 12 ^f^	39 ± 22	83 ± 40
**Bone**	3 ± 2 ^a,c^	4 ± 1 ^b,c^	1.1 ± 0.4 ^d,e^	2 ± 1 ^d,e^	5.0 ± 0.3 ^f,g^	7 ± 1 ^f^	9 ± 1 ^g^	11 ± 2

^a^ Significant difference (*p* < 0.05) between [^57^Co]Co and [^68^Ga]Ga. ^b^ Significant difference (*p* < 0.05) between [^57^Co]Co and [^111^In]In. ^c^ Significant difference (*p* < 0.05) between [^57^Co]Co and [^125^I]I. ^d^ Significant difference (*p* < 0.05) between [^68^Ga]Ga and [^111^In]In. ^e^ Significant difference (*p* < 0.05) between [^68^Ga]Ga and [^125^I]I. ^f^ Significant difference (*p* < 0.05) between [^111^In]In and [^125^I]I. ^g^ Significant difference between *N*- and *C*-terminal placement of the label (unpaired *t*-test).

**Table 8:** Tumor-to-normal-tissue ratios for DARPin Ec1 variants labeled with [^57^Co]Co, [^111^In]In or [^125^I]I-HPEM at *N*- or *C*-terminus 24 h p.i. in Balb/c nu/nu mice bearing EpCAM-expressing DU145 xenografts (average from 4 mice ± SD). One-way ANOVA with Bonferroni’s multiple comparisons test was performed to find significant differences between Ec1 variants with the same label position (*N*- or *C*-terminus).

	[^57^Co]Co N	[^57^Co]Co C	[^68^Ga]Ga N	[^68^Ga]Ga C	[^111^In]In N	[^111^In]In C	[^125^I]I N	[^125^I]I C
**Blood**	29 ± 8 ^a^	59 ± 32 ^a^	64 ± 19 ^c,d^	182 ± 64 ^c^	11 ± 4 ^d^	68 ± 12	29 ± 8 ^a^	59 ± 32 ^a^
**Sal. gland**	6 ± 2 ^b^	5 ± 2 ^b^	5 ± 2 ^c^	9 ± 4 ^c^	36 ± 16 ^d^	163 ± 54	6 ± 2 ^b^	5 ± 2 ^b^
**Lung**	11 ± 3	13 ± 5 ^b^	14 ± 5 ^d^	24 ± 3 ^c^	20 ± 7 ^d^	70 ± 29	11 ± 3	13 ± 5 ^b^
**Liver**	0.4 ± 0.1 ^b^	0.5 ± 0.2 ^b^	0.6 ± 0.1 ^c,d^	1.5 ± 0.4 ^c^	5 ± 2 ^d^	15 ± 3	0.4 ± 0.1 ^b^	0.5 ± 0.2 ^b^
**Spleen**	1.6 ± 0.4 ^b^	2 ± 1 ^b^	2 ± 1 ^c,d^	5 ± 1 ^c^	19 ± 7 ^d^	41 ± 5	1.6 ± 0.4 ^b^	2 ± 1 ^b^
**Stomach**	9 ± 2 ^b^	7 ± 3 ^b^	8 ± 3 ^c,d^	14 ± 2 ^c^	33 ± 13	59 ± 22	9 ± 2 ^b^	7 ± 3 ^b^
**Kidney**	0.02 ± 0.01 ^b^	0.02 ± 0.01 ^b^	0.03 ± 0.01 ^c^	0.04 ± 0.02 ^c^	3 ± 1 ^d^	13 ± 1	0.02 ± 0.01 ^b^	0.02 ± 0.01 ^b^
**Muscle**	25 ± 7 ^b^	17 ± 5 ^b^	24 ± 1 ^c^	28 ± 6 ^c^	132 ± 49	189 ± 55	25 ± 7 ^b^	17 ± 5 ^b^
**Bone**	3 ± 1	3 ± 1	5 ± 1	7 ± 2	7 ± 4	6 ± 3	3 ± 1	3 ± 1

^a^ Significant difference (*p* < 0.05) between [^57^Co]Co and [^111^In]In. ^b^ Significant difference (*p* < 0.05) between [^57^Co]Co and [^125^I]I. ^c^ Significant difference (*p* < 0.05) between [^111^In]In and [^125^I]I. ^d^ Significant difference (*p* < 0.05) between *N*- and *C*-terminal placement of the label (unpaired *t*-test).

## Data Availability

The data generated during the current study are available from the corresponding authors upon reasonable request.

## Supplementary Materials

The following are available online at https://www.mdpi.com/article/10.3390/cancers13143589/s1, Figure S1: ESI-MS spectra of Ec1 variants conjugated to DOTA chelator at *N*- or *C*-terminus. Calculated molecular weight for both variants 19140.9, found 19141.7 for (A) N-terminal DOTA-CE_3_-Ec1-(HE)_3_ and 19141.7 for (B) *C*-terminal (HE)_3_-CE_3_-Ec1-DOTA, Figure S2: SDS-PAGE analysis of (A) [^57^Co]Co-DOTA–Ec1 and [^57^Co]Co-Ec1–DOTA, and (B) [^68^Ga]Ga-DOTA–Ec1 and [^68^Ga]Ga-Ec1–DOTA. [^57^Co]Co-EDTA or [^68^Ga]Ga-citrate were used as markers for low-molecular-weight compounds, Figure S3: SDS-PAGE analysis of (A) [^111^In]In-DOTA–Ec1 and [^111^In]In-Ec1–DOTA, and (B) [^125^I]I-HPEM–Ec1 and [^125^I]I-Ec1–HPEM. [^111^In]In-EDTA or [^125^I]NaI were used as markers for low-molecular-weight compounds, Figure S4: Cellular processing of Ec1 variants radiolabeled with (A) [^125^I]I-HPEM, (B) [^111^In]In, (C) [^68^Ga]Ga and (D) [^57^Co]Co at *N*- or *C*-terminus, by DU145 prostate cancer cells during continuous incubation over 24 h (for [^68^Ga]Ga over 3 h). Data are presented as mean from three samples ± SD; error bars may not be visible when they are smaller than symbols, Figure S5: Overlay of cellular processing data for Ec1 variants radiolabeled with [^125^I]I-HPEM, [^111^In]In and [^57^Co]Co at *N*- or *C*-terminus, by DU145 prostate cancer cells during continuous incubation over 24 h. Data are presented as mean from three samples ± SD; error bars may not be visible when they are smaller than symbols.
