# Supplementary material for: Influence of the Position and Composition of Radiometals and Radioiodine Labels on Imaging of Epcam Expression in Prostate Cancer Model Using the DARPin Ec1
Source: Cancers (Basel). 2021 Jul 17;13(14):3589. doi: 10.3390/cancers13143589 (PMC8304184; doi:10.3390/cancers13143589)
Supplement: Supplementary file 1 [file cancers-13-03589-s001.zip › cancers-1291329-supplementary.pdf]

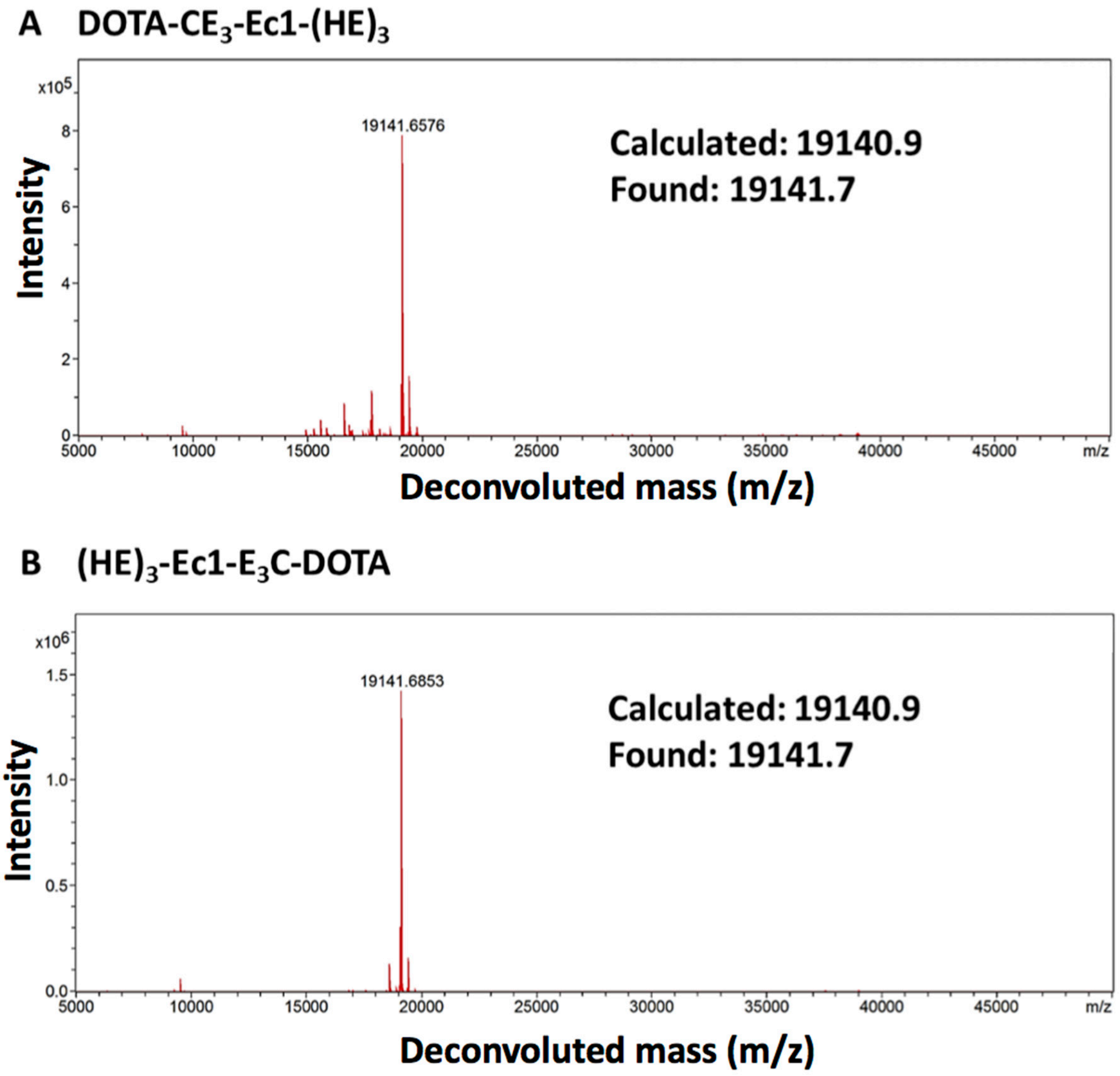

Supplementary Figure S1. ESI-MS spectra of Ec1 variants conjugated to DOTA chelator at N- or C-terminus. Calculated molecular weight for both variants 19140.9, found 19141.7 for (**A**) N-terminal DOTA-CE_3-Ec1-$(HE)_3$ and 19141.7 for (**B**) C-terminal $(HE)_3$-CE_3-Ec1-DOTA.

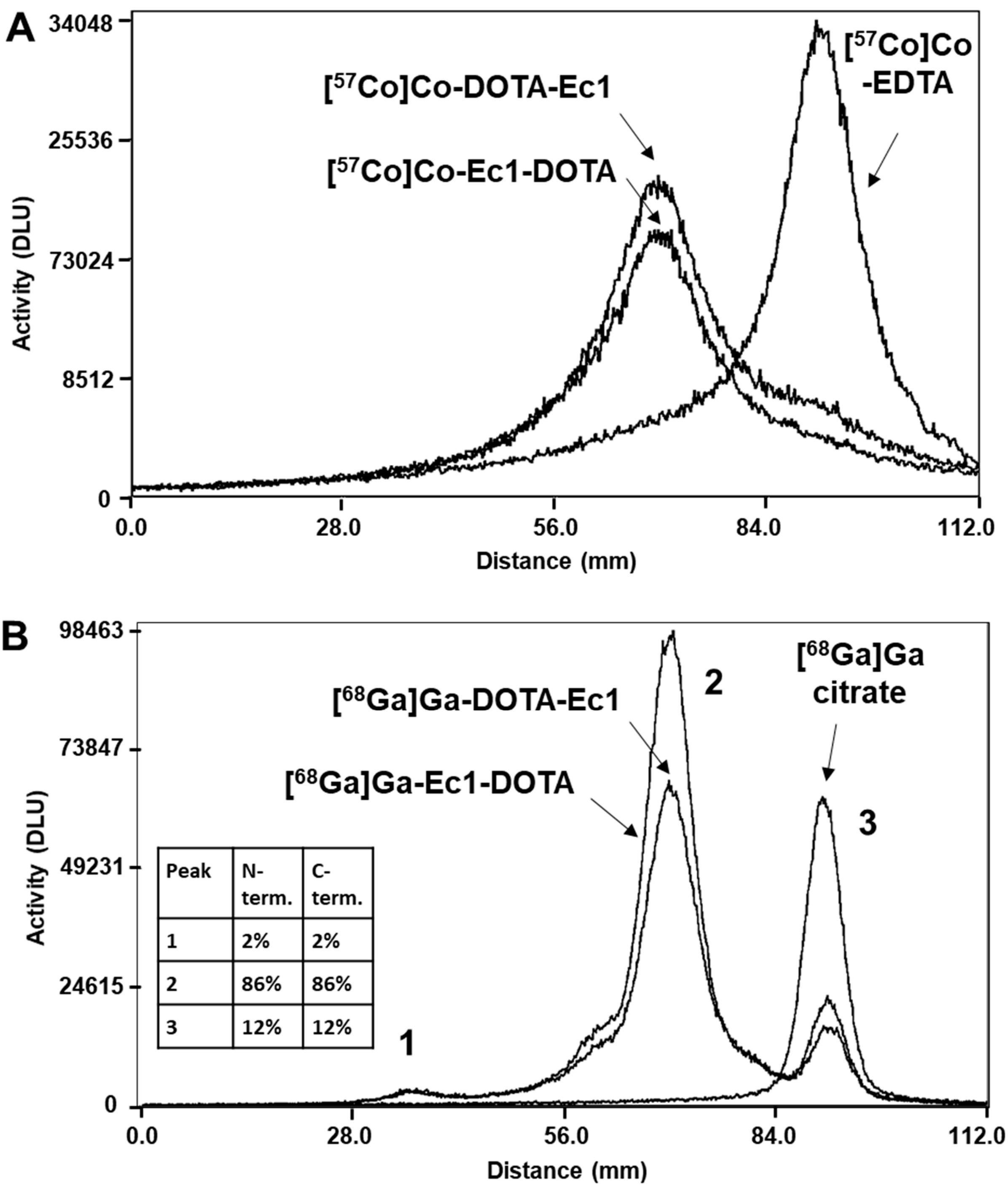

Peak	N-term.	C-term.
1	2%	2%
2	86%	86%
3	12%	12%

Supplementary Figure S2. SDS-PAGE analysis of (**A**) [^{57}Co]Co-DOTA-Ec1 and [^{57}Co]Co-Ec1-DOTA, and (**B**) [^{68}Ga]Ga-DOTA-Ec1 and [^{68}Ga]Ga-Ec1-DOTA. [^{57}Co]Co-EDTA or [^{68}Ga]Ga-citrate were used as markers for low molecular weight compounds.

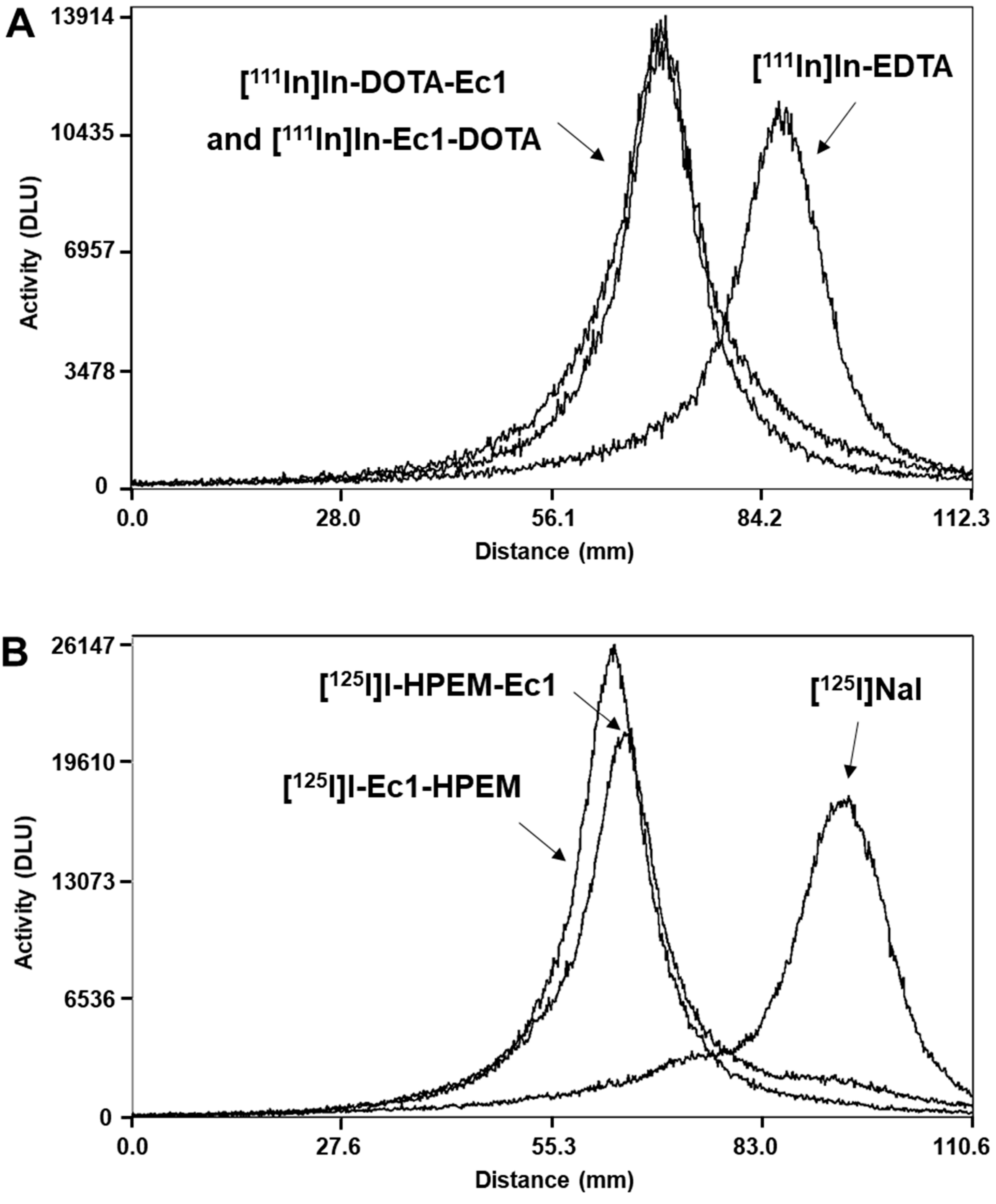

Supplementary Figure S3. SDS-PAGE analysis of (**A**) [^{111}In]In-DOTA-Ec1 and [^{111}In]In-Ec1-DOTA, and (**B**) [^{125}I]I-HPEM-Ec1 and [^{125}I]I-Ec1-HPEM. [^{111}In]In-EDTA or [^{125}I]NaI were used as markers for low molecular weight compounds.

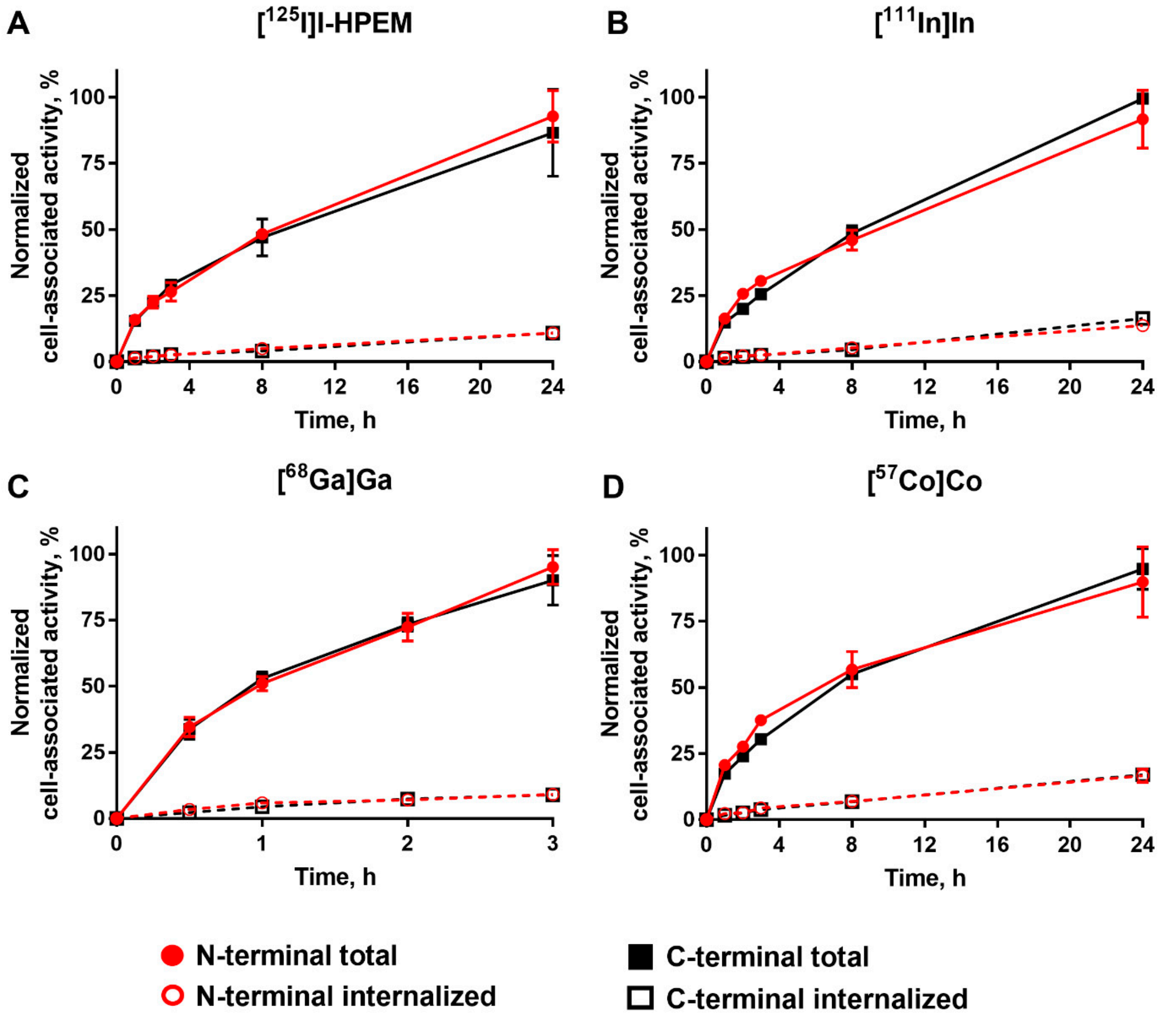

Figure S4. Cellular processing of Ec1 variants radiolabeled with (**A**) [^{125}I]I-HPEM, (**B**) [^{111}In]In, (**C**) [^{68}Ga]Ga and (**D**) [^{57}Co]Co at N- or C-terminus, by DU145 prostate cancer cells during continuous incubation over 24 h (for [^{68}Ga]Ga over 3 h). Data are presented as mean from three samples ± SD; error bars may not be visible when they are smaller than symbols.

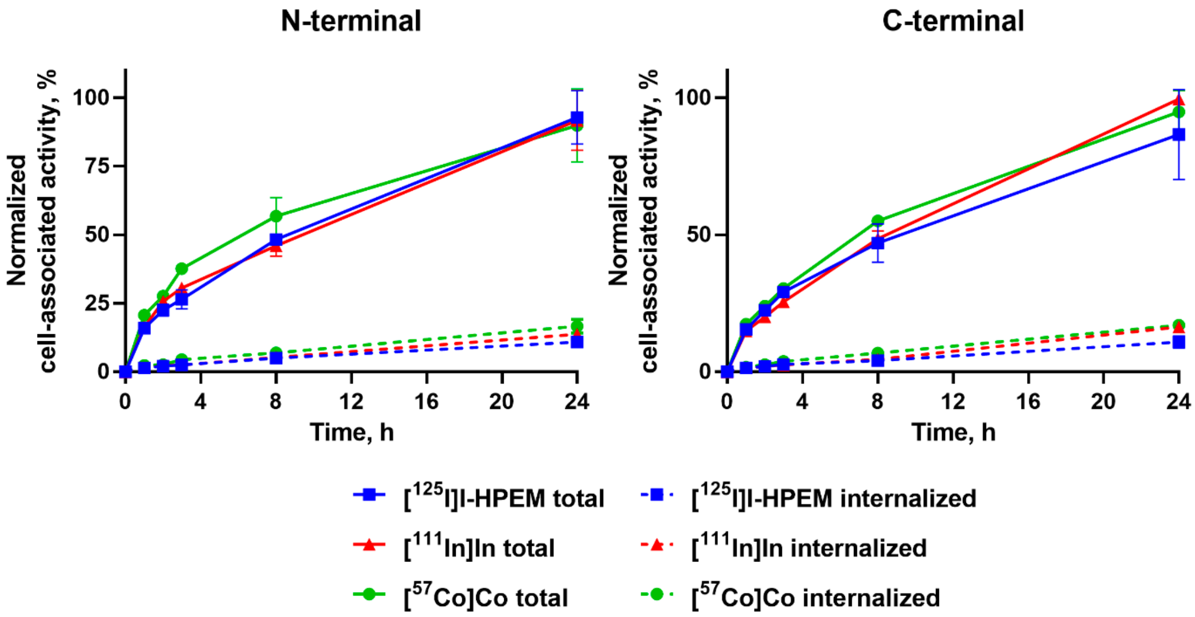

Figure S5. Overlay of cellular processing data for Ec1 variants radiolabeled with [^{125}I]I-HPEM, [^{111}In]In, and [^{57}Co]Co at N- or C-terminus, by DU145 prostate cancer cells during continuous incubation over 24 h. Data are presented as mean from three samples ± SD; error bars may not be visible when they are smaller than symbols.
